# Long-Term Oncological Control by Repeated Minimally Invasive Hepatectomy for Intrahepatic Cholangiocarcinoma Exhibiting Atypical Marker Expression of CK7-CK20+: A Case Report

**DOI:** 10.7759/cureus.50639

**Published:** 2023-12-16

**Authors:** Kei Harada, Takahisa Fujikawa, Yusuke Uemoto, Taisuke Matsuoka

**Affiliations:** 1 Surgery, Kokura Memorial Hospital, Kitakyushu, JPN

**Keywords:** cytokeratin 20, cytokeratin 7, robotic liver resection, minimally invasive hepatectomy, intrahepatic cholangiocarcinoma

## Abstract

There are few reports of repeated liver resections being performed multiple times for intrahepatic recurrence of intrahepatic cholangiocarcinoma (ICC). We performed five minimally invasive liver resections and two minimally invasive lung resections for ICC with metachronous intrahepatic recurrence and lung metastases. Pathological examination revealed that all resected tumors were moderately differentiated mass-forming ICC with immunohistochemical marker expression of CK7 negative and CK20 positive. We present this as a rare case of ICC with atypical marker expression in which long-term tumor control was achieved with multiple minimally invasive liver resections over 47 months from the initial diagnosis.

## Introduction

The only treatment that can be expected to be curative for intrahepatic cholangiocarcinoma (ICC) is liver resection, but postoperative recurrence has been reported to be as high as 60%, and the five-year survival rate after liver resection is about 35% [1,2]. The mode of recurrence is frequently accompanied by multiple recurrences in the remnant liver and distant metastasis to other organs, such as lymph nodes [3], and the indications for repeat hepatectomy are considered limited. On the other hand, there are reports that the prognosis is relatively good in cases where a repeat hepatectomy is performed for a single recurrence of the residual liver without distant metastasis to other organs [4].

Recently, it has been reported that immunostaining for cytokeratin 7 (CK7), CK20, and CDX2 can help tell the difference between ICCs and metastatic liver tumors [5]. The majority of ICCs are positive for CK7 and either negative or weakly positive for CK20. On the other hand, only four percent of ICC cases had CK7-/CK20+ [6], in which the prognosis or appropriate therapeutic modality remains unknown.

Herein, we present the rare case of ICC with atypical immunohistochemical marker expression of CK7-/CK20+, in which a relatively good prognosis was achieved by undergoing repeat minimally invasive hepatectomy and partial lung resection for five metachronous ICC recurrences that occurred after curative resection.

## Case presentation

**Figure 1:**
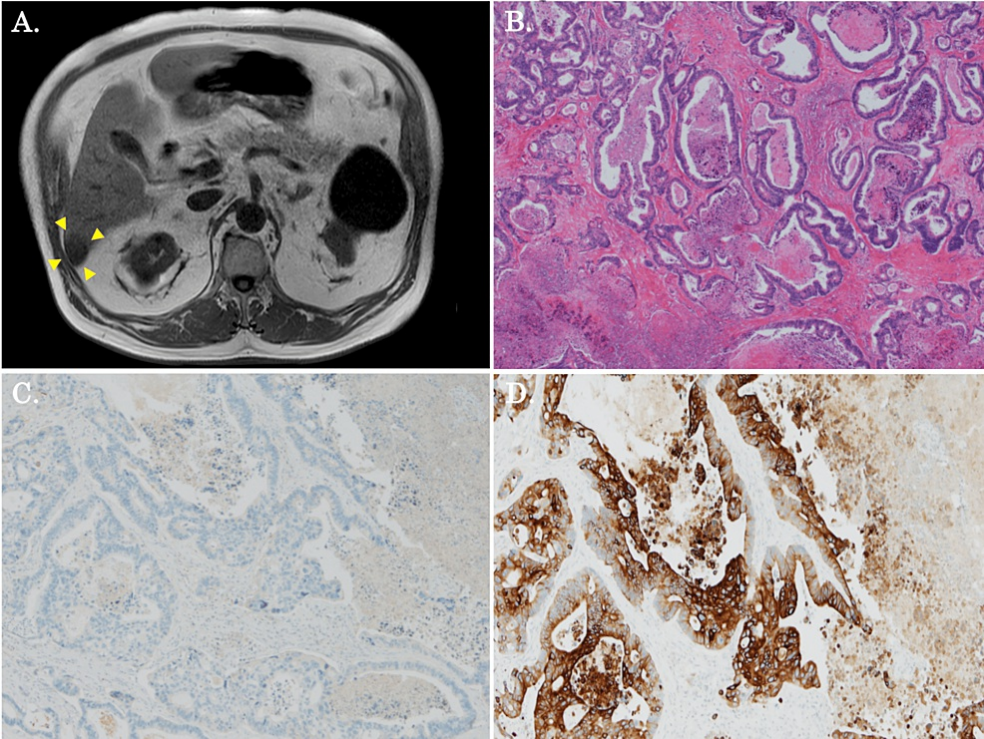
MRI findings prior to laparoscopic S6 partial liver resection (the first operation) A: MRI showed that the liver tumor in S6 was 30 mm in size (yellow arrowheads). B: Pathological findings were moderately differentiated adenocarcinoma with no capsular invasion or vascular invasion. C, D: Immunological staining results showed a CK7-/CK20+ pattern (C; CK7, D; CK20).

A 78-year-old man was referred to our hospital for treatment of a liver segment 6 (S6) tumor. A computed tomography (CT) scan showed a 30 mm-sized liver tumor in the S6. The patient was taking a direct oral anticoagulant for chronic atrial fibrillation and had type 2 diabetes and stage G5 chronic renal failure. He had no chronic liver diseases due to hepatitis B virus and hepatitis C virus infections. For the S6 tumor, liver contrast magnetic resonance imaging (MRI) revealed T1 weighted imaging (T1WI) low signal, T2WI high signal, and diffusion-weighted imaging (DWI) high signal, as well as decreased Fe uptake (Figure 1A). Gastrointestinal screening and ^18^F-fluorodeoxyglucose positron emission tomography (FDG-PET) were performed to look for the primary tumor, but the primary tumor was unknown. Initial tumor marker levels were as follows: carcinoembryonic antigen (CEA), 185.5 ng/ml; carbohydrate antigen 19-9 (CA19-9), 105 ng/ml; alpha-fetoprotein (AFP), 1.1 ng/ml; protein induced by vitamin K absence or antagonist-II (PIVKA-II), 10.7 mAU/ml. From the above results, ICC or metastatic liver tumor was suspected. A laparoscopic S6 partial liver resection was performed as a non-anatomic liver resection with a surgical margin of one cm from the tumor. The pathological findings of the resected liver tumor were CK7-/CK20+, moderately differentiated, mass-forming ICC (Figure 1B-1D).

**Figure 2:**
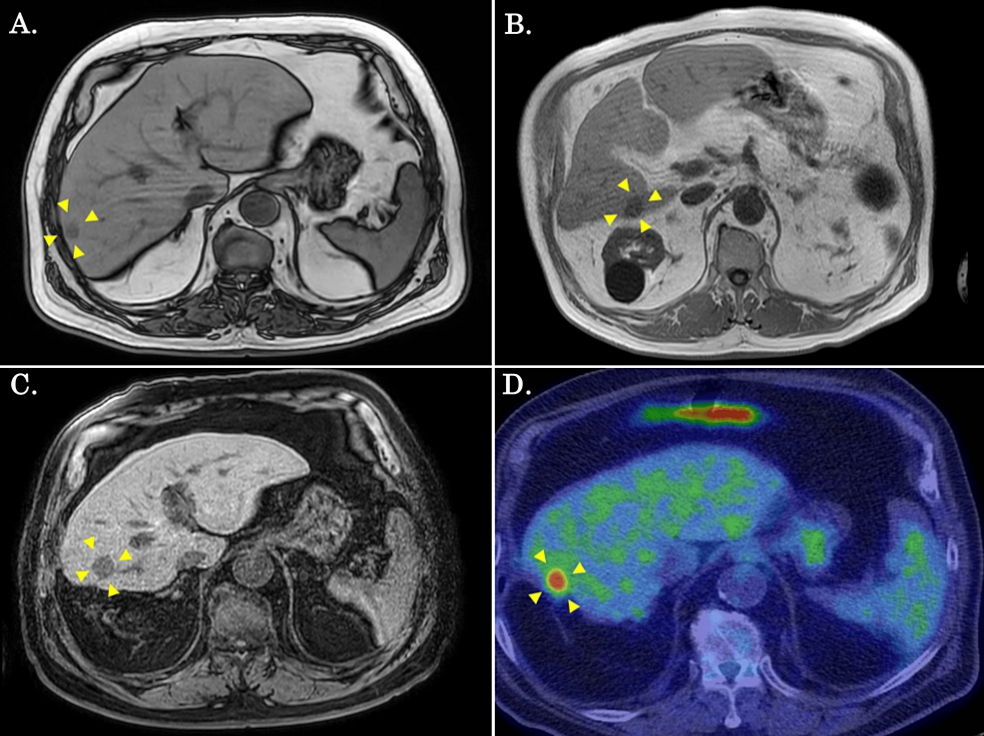
Preoperative findings at the following repeated liver resection in the current case A: MRI findings prior to laparoscopic S7/6 partial liver resection (the second operation). B: MRI findings prior to robotic S6 partial liver resection (the third operation). C: MRI findings prior to robotic S6 subsectionectomy (the fourth operation). D: FDG-PET findings prior to robotic S7 partial liver resection (the sixth operation). Arrowheads in each figure demonstrated the tumor. FDG-PET - ^18^F-fluorodeoxyglucose positron emission tomography

Twelve months after the initial surgery, CT revealed a two cm-sized mass at S7/6 of the remnant liver (Figure 2A). Surgery was considered appropriate because there were no problems with the patient's general condition, and no extrahepatic lesions were found. The tumor was removed by laparoscopic S7/6 partial liver resection. Histopathologically, the resected tumor was a two cm-sized moderately differentiated adenocarcinoma with no vascular invasion. Cancer was not found in the surgical margin. The histology was similar to that of the initial resection. Chemotherapy was not administered postoperatively due to severe renal dysfunction and at the patient's request.

A two cm-sized mass in S6 of the residual liver was discovered by MRI thirteen months after the second surgery (Figure 2B). There were no extrahepatic lesions, and it was determined that this was a single intrahepatic recurrence of ICC. A robotic S6 partial liver resection was performed because the patient's overall condition was good. The resected tumor was a moderately differentiated adenocarcinoma, according to histopathology. Immunohistochemistry revealed CK7-, CK20+, CK19+, CDX2-, and hepatocyte-. Furthermore, MRI revealed a 1.5 cm-sized single mass in the S6 five months after the third surgery (Figure 2C), and based on the same considerations, a robotic S6 subsectionectomy of the liver was performed.

**Figure 3:**
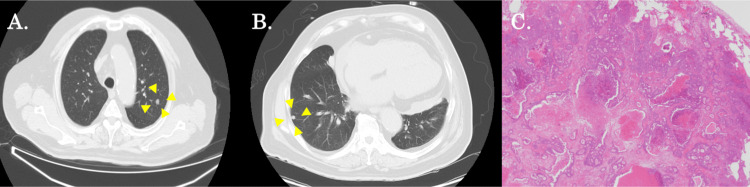
CT findings prior to thoracoscopic partial lung resection (the fifth operation) and pathological findings of the resected specimen A, B: CT scans showed that the tumors at the left upper lobe (A) and at the right lower lobe (B) were seven mm and eight mm in size, respectively (yellow arrowheads). C: It was a moderately differentiated adenocarcinoma with pathological findings similar to the initial liver resection specimen.

Afterward, CT scans revealed a single 1.5 cm-sized mass in the right and left lungs four months after the fourth surgery (Figure 3A, 3B). He received a thoracoscopic partial resection of the left upper lobe and the right lower lobe, as he was suspected of having a metastatic lung tumor. The resected tumors consisted of moderately differentiated adenocarcinoma with a histological appearance similar to a previously resected specimen, and the findings were consistent with a metastatic lung tumor (Figure 3C). Furthermore, seven months after the fifth surgery, a CT scan and FDG-PET revealed a two cm-sized mass at S7 of the liver (Figure 2D), and a robotic S7 partial liver resection was performed. The resected tumor was moderately differentiated adenocarcinoma with no vascular invasion, and the resection margins were cancer-free. The histology was the same as it had been at the time of the initial resection. No gallbladder removals have been performed to date.

**Figure 4:**
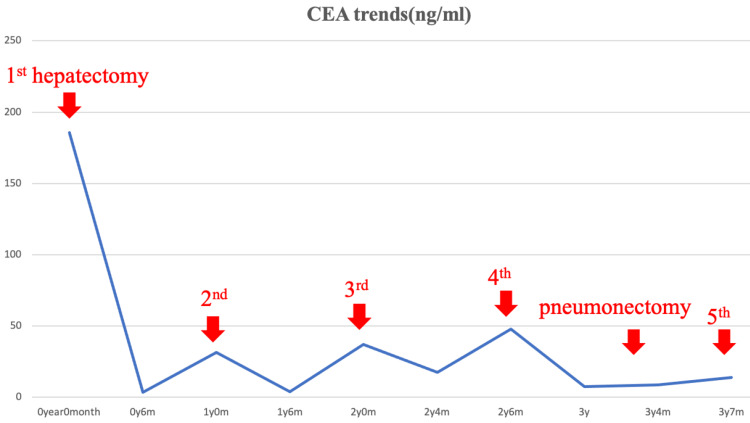
Following the first surgery, tumor markers related to CEA decreased with tumor resection and increased with tumor recurrence CEA - carcinoembryonic antigen

His tumor markers related to CEA decreased concurrently with tumor resection following the initial surgery and increased concurrently with tumor recurrence (Figure 4), and currently, CEA values are maintained without increasing. Thanks to the minimally invasive surgical intervention using robotic or endoscopic surgery, the postoperative recovery went without any incident during the whole therapeutic process, and his renal function was maintained without the requirement of hemodialysis. The patient is still doing well and free from oncological therapy without any recurrence four months after the last operation (47 months after the initial diagnosis).

## Discussion

ICC is a rare tumor that accounts for approximately 6.9% of primary liver cancers [7]. ICC is visually classified into three types: mass-forming, bile duct invasion, and bile duct growth, but surgical resection is the only definitive treatment regardless of the macroscopic type [8]. However, the prognosis is generally poor, and even after radical resection, the five-year postoperative survival rate is 44.9% [1]. Furthermore, residual liver recurrence, which is the most common type of recurrence, is said to be multiple or to be accompanied by recurrence in other organs such as lymph nodes, lungs, and peritoneum [1,2]. Compared to patients who received chemotherapy, transarterial chemoembolization, selective internal radiotherapy, radiofrequency ablation, or supportive care, patients who underwent repeated hepatectomy for recurrent ICC had a significantly longer overall survival, according to Bartsch et al. [9]. However, there are few reports of repeated hepatectomy for intrahepatic recurrence, as in our case. Furthermore, most guidelines do not address strategies for recurrence after R0 resection of ICC.

In our case, there were no extrahepatic lesions at the time of surgery, the patient's liver reserve was good, and his general condition was stable, so surgery was judged appropriate. Following the first surgery, tumor markers related to CEA decreased with tumor resection and increased with tumor recurrence. CA19-9, AFP, and PIVKA-II levels were all consistently within normal ranges. Histopathological examination revealed that, as in the first surgery, all four tumors in the re-hepatectomy specimens were moderately differentiated adenocarcinomas with no capsular invasion or vascular invasion. The resection margins were all cancer-free in all cases. Furthermore, no hepatocellular carcinoma components were found in the tumor area. An additional immunostaining test revealed that CK7 was negative and CK20 was positive. The third re-hepatectomy specimen revealed CDX2- and hepatocyte-, and was negative for mixed liver cancer and bile duct cell carcinoma.

The CDX2 gene is required for the development and differentiation of intestinal epithelial cells. Because CDX2 is expressed from the duodenum to the rectum, it is an extremely sensitive marker for identifying small and large intestine tumors [5]. In our case, the patient was CDX2 negative, and gastrointestinal screening and FDG-PET revealed no gastrointestinal tumor, ruling out a metastatic liver tumor. The histopathological and immunohistochemical findings of the re-hepatectomized lesions are similar, as is the time to recurrence, which ranges from six to 13 months. Overall, we concluded that the clinical course was consistent with the metachronous intrahepatic recurrence of mass-forming ICC.

Cytokeratin is one of the intermediate filament proteins that make up the cytoskeleton within epithelial cells, and it is classified into CK1-CK20 based on molecular weight. The expression patterns of CK7 and CK20 can be used to predict the primary site of adenocarcinoma [10]. Sixty-four percent of intrahepatic cholangiocarcinoma exhibit a CK7+/CK20+ staining pattern, while 31% exhibit a CK7+/CK20- staining pattern, according to Rullier et al. [6]. Furthermore, according to Chu et al., 43% of cholangiocarcinomas are CK7+/CK20+, 50% are CK7+/CK20-, and CK7-/CK20+ is 0% [11]. CK7-/CK20+ staining pattern is said to be very common in colorectal carcinoma and Merkel cell tumor. In our case, it was discovered to be CK7-/CK20+, a very unusual result. According to research by Liu et al., a high expression level of CK7 in ICC indicates a poor prognosis and an integrated analysis of their expression is a reliable indicator to assess patient outcomes [12]. It was suggested that repeated hepatectomy might help to extend the prognosis in the case of a relatively slow-invasive tumor with CK7- marker expression, like in the current case.

In our case, we were able to perform five repeat hepatectomy because the patient's general condition and liver reserve were good each time the surgery was performed, but also because there was no extrahepatic metastasis and no recurrence. Multiple surgeries were also made possible by reducing surgical invasiveness through laparoscopic and robotic liver resection. According to Nakajima et al., in four cases where hepatectomy was performed twice or more for residual liver recurrence due to ICC, the average duration from the initial surgery to the first intrahepatic recurrence was 21 (15-25) months [13]. In that report, the pathology of the initial liver tumor was moderately differentiated adenocarcinoma in three of the four cases, and the ICC was of the mass-forming type in all cases, and it was determined that repeated hepatectomy would contribute to disease control in each case [13]. On the other hand, throughout the clinical course of these patients, postoperative chemotherapy was not administered due to chronic renal failure, and the patient didn't wish for it. We think that additional research and consideration of postoperative chemotherapy is required [14,15].

## Conclusions

We present a rare case of CK7-/CK20+ atypical ICC that was treated for 47 months after the initial diagnosis, with long-term tumor control achieved through repeated minimally invasive hepatectomy. In the case of ICC recurrence with atypical immunostaining marker expression, as in our case, repeated minimally invasive hepatectomy may contribute to a longer prognosis. It was also suggested that strict postoperative follow-up, appropriate selection and analysis of cases in which the effects of surgical treatment can be expected, and minimizing any surgical invasiveness are all important.
